# Opportunistic screening for iron-deficiency in 6–36 month old children presenting to the paediatric emergency department

**DOI:** 10.1186/1471-2431-5-42

**Published:** 2005-11-22

**Authors:** Martin V Pusic, Brenda J Dawyduk, David Mitchell

**Affiliations:** 1 University of B.C., Dept of Pediatrics, Vancouver, BC Canada; 2 University of Manitoba, Winnipeg, Manitoba, Canada; 3 McGill University, Dept. of Pediatrics, Montreal, Quebec, Canada

## Abstract

**Background:**

The Complete Blood Count (CBC) is a test frequently performed on children presenting to the Pediatric Emergency Department (PED), usually for the evaluation of an infectious illness. The CBC also allows for screening for Iron-deficiency Anemia. This study aims to determine the prevalence of a low Mean Cell Volume (MCV) in children having a CBC performed during a PED visit and whether physicians acted upon the abnormal value.

**Methods:**

We present a retrospective cohort study. We reviewed the PED charts of all children 6–36 months of age who had a CBC performed during a 4-month period and the red blood cell mean cell volume was low. Our main outcome variable was whether or not the possible iron deficiency was addressed through documentation of either iron therapy or further investigation.

**Results:**

938 children had a CBC performed during the two periods. Of these, 78 (8%) had an abnormal MCV or Hemoglobin with no previously identified explanation. Physicians documented either treatment or follow-up investigations in 27 cases (35%, 95% CI: 24–46%). Factors associated with the physician documenting either treatment or investigation plan were the following: hemoglobin level (OR 12.6; 95%CI: 4.0, 39) and age ≤ 18 months (OR 4.2; 95%CI: 1.4, 13).

**Conclusion:**

Children who have had a CBC in the PED can be screened for iron deficiency at no additional cost. Physicians may be under-utilizing this information.

## Background

Iron deficiency anemia in infants and young children can have serious adverse effects on their development including irreversible cognitive impairment and behaviour problems [1-5]. The negative sequelae can be prevented with simple treatment with iron-fortified infant formula or oral iron supplementation. High risk groups include children from low socioeconomic status families, Chinese children, aboriginal children, infants of low birth weight, children whose mothers were iron-deficient during pregnancy, and children who consume cow's milk [6,7]. Despite an overall decline in the incidence over the past 20 to 30 years there is still a significant proportion of children at risk. The prevalence of iron-deficiency anemia among a random group of Canadian 9 month olds was 7%, and 24% had low iron stores, despite the introduction of iron-fortified cereals by six months of age [7]. Among African-American children aged 9 to 36 months the prevalence of iron deficiency anemia was 8% [8].

The Canadian Task Force on Preventive Health Care provides a grade B recommendation (fair evidence to support) for routine hemoglobin measurement for infants at high risk [6]. The U.S. Preventive Services Task Force recommends that screening should be offered once to all infants, and the American Academy of Pediatrics recommends at least one measurement of hemoglobin or hematocrit in infancy, and at least during the ages of 1 to 4 years and again in adolescence [9].

Basic screening for iron-deficiency anemia consists of a complete blood count (CBC) looking for hemoglobin less than the age-adjusted normal range, with a sensitivity of 86% and a specificity of 97% [6]. The diagnosis is supported by a low mean corpuscular volume (MCV), and increased red cell distribution width (RDW). Confirmation of the diagnosis is commonly obtained with a trial of iron supplementation and measurement of the response [10]. More complex measures of iron stores including serum ferritin, transferring saturation and erythrocyte protoporphyrin activity are used to more accurately determine iron status [11].

It is unclear what proportion of infants and children are currently being screened as recommended. Ideally every child would be screened but this likely does not happen for reasons of cost and the pain associated with the blood test.

A large number of infants and children who are assessed in emergency departments have a CBC done. This presents an opportunity to screen for iron-deficiency anemia. However, in this setting the physician is likely to be focused on the white cell count as most of these patients are being assessed for the possibility of infection. As a result, CBC indices that suggest iron-deficiency anemia may be overlooked. Our hypothesis was that the CBC information that pertains to iron-deficiency is underutilized given that this is not usually the primary indication for the test. If confirmed, we proposed to raise physician awareness of this method of secondary screening.

## Methods

### Setting

The Montreal Children's Hospital is a 181-bed tertiary-care referral center affiliated with McGill University. The Pediatric Emergency Department (PED) has an annual census of 85,000 visits and is staffed by 6 full-time Pediatric Emergency Medicine physicians. A large group of community-based pediatricians also contribute from 1 to 6 shifts per month. Medical students, paediatric, family medicine and emergency medicine residents and fellows all do training rotations in the PED.

### Study design

This is a retrospective before-after cohort study. We identified all children who had a Complete Blood Count (CBC) performed in the PED during a 2-month period (February–March 1999), implemented an educational intervention and then collected data during the same two months a year later. The Institutional Review Board approved the study.

### Subjects

Using our laboratory information system, we were able to identify all children who had a CBC done in the PED during the two study periods. We then selected children aged 6 to 36 months based on their age at the time the CBC was done. We excluded children with a known history of the following: anemia on treatment, sickle cell disease or trait, thalassaemia or trait, chronic illness likely to cause anemia. Patients who presented twice during a study period were handled as follows: if, on the first visit iron-deficiency was recognized, they were excluded on the next visit. If, on the first visit, iron-deficiency was not identified, then they were included on the second visit.

### Laboratory techniques

CBCs were determined using a Bayer Advia 120 hematology analyzer (Bayer AG, Leverkusen, Germany). We defined the lower limit of normal for MCV as 70 fl while 110 g/L was the cutoff for normal Hemoglobin.

### Definitions

#### MCV addressed

We identified children as having had their iron status addressed if either treatment or further investigations was documented on the PED encounter sheet. Treatment could include any iron preparation initiated by the PED physician. Further assessment could include specific referral to their family doctor for assessment of iron deficiency anemia or arrangement of confirmatory laboratory tests (any of: Hgb Electrophoresis, Serum Fe, Ferritin, Free Erythrocyte Protoporphyrin, Transferrin Level).

#### MCV not addressed

Children with a low MCV who did not have either treatment or arrangements for further assessment, as defined above, documented on either their PED encounter sheet or, if admitted, on their discharge summary.

### Data collection

From the Laboratory Information System we collected the hemoglobin measurement, all standard CBC indices, ordering physician name, patient date of birth and hospital chart number. We performed chart reviews on all children with low MCV. We limited the chart review to the PED encounter sheet of the CBC date as well as the inpatient chart for those patients who were admitted at the time of the initial encounter. From the charts we collected information concerning the reason for the visit, any past history relevant to anemia, a list of medications, and whether any note had been made regarding possible iron-deficiency.

### Educational intervention

We collected the data in two sessions. In April 1999 we identified the first cohort (Feb–Mar 1999). These initial data were analyzed and showed that a significant number of patients were not having possible iron deficiency addressed. We presented this information (that we pediatricians were missing up to 75% of children with abnormal MCV's in this age group) in a one-hour PED Grand Round in June 2000. To reinforce the message, we mailed a one-page abstract to them with their monthly work schedules in January 2000. This abstract stressed the importance of examining the MCV results on all CBC's done. We collected the second set of data after these interventions in Feb-March 2000. We hoped that this intervention would improve the rate of recognition of abnormal MCVs.

### Data analysis

We analyzed the data using descriptive statistics and used chi-squared tests to look for factors that differed between groups where the possible iron-deficiency had been addressed. We calculated 95% confidence intervals around proportions using the binomial distribution. We also developed a univariate logistic regression model to see which factors predict proper identification and documentation of iron deficiency. Our sample size was determined based on the following assumptions: with 25 subjects in each group, we could detect an improvement from 10% identification to 50% with a power of 80% at alpha = 0.05.

## Results

### PED patients with CBCs

During the four months studied, a total of 7571 patients aged 6–36 months were seen in the PED. Of these, 934 (12%) had CBC's performed which is 29% of the 3206 CBCs performed for all children seen in the PED during the study periods.

**Figure 1:**
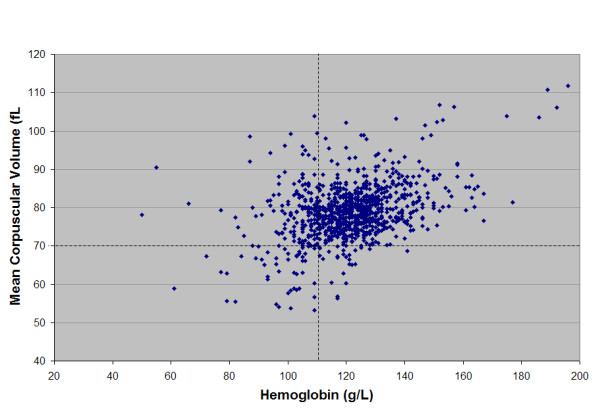
MCV versus Hemoglobin for all children having a CBC in the PED.

The MCV was less than 70 fl in 94 patients (10%) while 184 (19.7%) of the children had a hemoglobin < 110 g/L. Fifty-seven had both low MCV and low hemoglobin. The relationship between MCV and HgB is shown graphically in Figure 1. Children 18–36 months of age did not have significantly different MCV (79.4 fl) or hemoglobin (122 g/L) values compared with children 6–17 months (78.5 fl, 124 g/L respectively; both p = NS).

### Patients with possible iron deficiency

Of the patients with low MCV, 16 were excluded from the analysis for the following reasons: sickle cell disease (1), thalassemia (3), previously identified iron deficiency (8) and chronic disease (2 – autoimmune hepatitis and liver mass). Two charts were missing. Discharge diagnosis and some form of discharge plan had been documented on every chart. For the 78 included patients, 45 (57%) had a hemoglobin value less than 110 g/L.

Patients' discharge diagnoses were available for all included patients. The most frequent diagnoses were: Gastroenteritis (19; 24%), Respiratory Illnesses (18; 23%), Nonspecific Viral Illness (13; 17%), and Febrile Seizures (4; 5%). No other diagnosis made up more than three of the cases.

Patients with low MCV's had an iron medication prescribed in 21 cases (27%). In another 6 (8%) cases, explicit follow-up was arranged so that, in total 27 of 78 patients had their low MCV addressed (35%; 95% CI: 24–46%). Even markedly decreased MCV's (≤65 fl) were addressed only 12 of 28 times (43%; 95% CI: 24–63%). Iron was addressed in 18/25 patients whose Hgb < 100.

### Regression model

In our univariate logistic regression model (Table 2), patient age and the value of the Hemoglobin measurement predicted that the iron status would be addressed. The following variables were not significantly associated: patient has a primary care physician, presence of fever, intravenous placement, admission to hospital, full-time versus part-time status of ordering Emergency Physician. In addition, there was no improvement in our rate of identifying children after our educational interventions.

**Table 1:** Assessment And Treatment Of Children Who Had A CBC Performed In The PED That Showed MCV < 70 fl.

	**N**	**Iron Therapy Prescribed**	**No Iron Therapy but Explicit Follow-Up Arranged**	**Neither Iron Therapy nor Follow-up Documented**
Hemoglobin Normal MCV Low	33	2	1	30
Hemoglobin Low MCV Low	45	19	5	21
All with low MCV	78	21	6	51

Hemoglobin cutoff 110 g/L; MCV (mean cell volume) cutoff 70 fL

**Table 2:** Factors Potentially Associated with Identification of Potential Iron Deficiency in Children with MCV <70

		**Total N**	**Number in whom Iron Prescribed or Tests Arranged**	**Odds Ratio**	**95% Confidence Interval**
Age	6–18 months	48	22	4.2	1.4, 13
	19–36 months	30	5		

Febrile	Yes	47	18	1.2	0.4, 3.4
	No	20	7		

IV Placed	Yes	43	15	1.0	0.4, 2.6
	No	35	12		

Admitted	Yes	14	8	3.2	1.0, 10
	No	64	19		

Hemoglobin	>100	53	9	12.6	4.0, 39
	≤99	25	18		
7						
Full-time PEM	Yes	46	19	0.63	0.2, 1.7
Attending Physician	No	26	8		
Educational	Pre	49	14	2.0	0.8, 5.3
Interventions	Post	29	13		

## Discussion

We report the results of a cohort study examining the prevalence of an index of iron deficiency (MCV) in children who have had a blood test in the PED. We found that PED physicians did not reliably document either a diagnostic or therapeutic plan to deal with the possibility of iron deficiency suggesting that this opportunity for secondary screening is being missed.

The risk of iron deficiency in U.S. children aged 12–35 months is approximately 9% [11]. Major national organizations such the American Academy of Pediatrics suggest that *all *children be screened with a blood test at age 12 months [9]. This is not commonly done possibly due to the perception that the risk of problems due to iron deficiency, for any individual child, are low especially when the diet includes iron-fortified foods. In addition, the cost, both financial and in terms of pain and inconvenience, is a barrier to routine screening.

The population of patients that present to urban PEDs is likely to be of low socio-economic status and skewed towards the preschool age group [12]. Both of these factors (low socio-economic status, age 3–24 months) are also predictive of iron deficiency suggesting that a PED-based secondary screening program could be successful.

For the PED patient population, we were unable to identify a published estimate of the predictive value of hemoglobin value or CBC indices compared with gold standard measures of iron status. Both low MCV and low Hemoglobin are imperfect predictors of iron deficiency. Using the National Health and Nutrition Examination Survey III (1988–1994) data, White showed that the positive predictive value of a low Hemoglobin value (<110 g/L) for iron deficiency is only 29% (95%CI: 20–38%) in children 12–35 months [11]. Iron deficiency was defined as the presence of ≥2 of the following: low ferritin, low transferrin saturation or high erythrocyte protoporphyrin.

Oski suggested a staging classification of iron status in which a period of iron-deficient erythropoiesis, characterized by low MCV and high RDW but normal hemoglobin, preceded frank anemia [3]. We felt that by identifying children at that stage, potential iron-deficiency could be intercepted at an early stage with simple interventions such as administration of an oral iron preparation [10]. Hemoglobin levels may be low due to recent viral illnesses or they may be elevated in dehydrated patients [10]. Interestingly, in our population, we found that more children had low hemoglobin values than low MCV's. We can only speculate that this might be due to the effects of intercurrent illness since we had no laboratory studies of iron stores available to us.

The health impact of mild iron deficiency is controversial. Anemia due to iron deficiency may be associated with behavioural changes, impaired psychomotor development and impaired cognitive function [13]. A frequently cited study found that children who were frankly anemic scored significantly lower on a test of cognitive function than controls [14]. The difference would not be perceptible in an individual child but would have important implications at the population level. Children with abnormal indices but normal hemoglobin did *not *differ from controls. This study and one other randomized controlled trial suggest that iron therapy can reverse the developmental effects after at least two months of therapy [13-15].

Widespread screening for thalassemia traits has not been routine likely because it is clinically benign, not of immediate clinical importance for young pediatric patients, and because they are concentrated within certain genetic backgrounds; however, discovering a thalassemia trait in a patient provides an opportunity for genetic counseling.

This is opportunistic secondary screening. The cost has already been paid and there is no need to change existing workflow to take advantage of this information in contrast to screening at the time of the well-child visit [16]. While it will likely only benefit those patients who have had a CBC done for another reason, heightened awareness of this issue might predispose PED physicians to address this issue more frequently in their patients. For example, increased screening of dietary histories in children at risk might be effective [17].

Our retrospective study is susceptible to a documentation bias. Physicians may well have considered iron deficiency but not documented it on the chart. This is all the more likely given that a health maintenance issue would rarely have been the primary indication for the visit to the PED. However, in informal discussions, our staff related that they were likely to be missing this diagnosis. Also, the absence of a Hawthorne effect in the publicized second phase of data collection suggests that the physicians do not consistently consider this diagnosis. We note that even if the documentation bias caused us to under-report appropriate therapy by a factor to two, we would still be missing close to half of the opportunities for closer assessment of these children.

Why do our physicians, many of whom are primary care pediatricians, not use this flagged information? The answer may lie in cognitive psychology. We speculate that a normally adaptive heuristic may be working against the physician in this particular situation. Heuristics are short-cuts that humans use to process information more quickly [18]. The CBC report presents the time-pressured PED physician a list of 12 numbers in a densely-typed column, all in the same font size. Some of these numbers are clearly more important than others: specifically, the White Blood Count, Hemoglobin and Platelet Counts. It may be that the physicians have subconsciously made a decision not to process all the numbers. Instead they have trained themselves to scan the list for only the three most important values. In essence, they see the list of numbers through an opaque mask that has only three holes cut out of it. This idea is supported by better performance of the physicians in detecting abnormal hemoglobin levels than abnormal MCVs.

Another heuristic is also likely working against the physician in this case. In the *search satisfying* heuristic, the physician who ordered a lab test for one reason (e.g. fever) will likely most closely attend to the feature of the result (e.g. White Blood Count) that is directly related to the reason for ordering the test. This may lead them to miss unanticipated results (e.g. anemia). What is not clear is why the physicians did not attend to the asterisks used to flag abnormal results (another adaptive heuristic is to scan a long list of lab reports for asterisks and then specifically attend to those results). Careful attention to the display of the information could increase physician attention to abnormal results without decreasing their efficiency [19].

We were disappointed that our educational interventions did not improve the situation. From the PED charts it appears that the rate at which clinicians arranged assessment or treated patients with evidence of iron deficiency did not change after we had presented a round to our Division and sent a one-page memo to every physician in the PED. These standard interventions have been shown to be relatively ineffective in changing physician behaviour in many settings [20]. While we did observe a trend towards improvment, it is likely instead that we will need to reanalyze and change our process of care [21]. For example, we could establish a protocol whereby all CBC samples that meet certain criteria for iron deficiency would automatically have a Free Erythrocyte Protoporphyrin test run on the same sample. The result would then be reported to the ordering physician. This strategy would have the advantage that, besides confirming the diagnosis, the added report would draw the physician's attention to the potential problem.

## Conclusion

In summary, we found that Pediatric Emergency Dept. physicians are not using all of the information available to them when they consider Complete Blood Count results. Careful attention to the Hemoglobin and MCV can suggest an opportunity for secondary prevention of iron-deficiency in children 6–36 months of age presenting to the ED. This information **c**omes at no cost to the patient and can help avoid cognitive impairment in some children.

## Abbreviations

CBC: Complete Blood Count

PED: Pediatric Emergency Department

MCV: Mean Cell Volume

## Competing interests

The author(s) declare that they have no competing interests.

## Authors' contributions

All authors participated in the design of the study. BD designed the data collection instrument, and with MP, did the chart reviews; DM and MP analyzed the data; MP drafted the manuscript with considerable revision by DM; All authors read and approved the final manuscript.

## Pre-publication history

The pre-publication history for this paper can be accessed here:


